# Osteoblast Derived Exosomes Alleviate Radiation- Induced Hematopoietic Injury

**DOI:** 10.3389/fbioe.2022.850303

**Published:** 2022-04-21

**Authors:** Jianqi Xue, Ruikai Du, Shukuan Ling, Jinping Song, Xinxin Yuan, Caizhi Liu, Weijia Sun, Yuheng Li, Guohui Zhong, Yinbo Wang, Guodong Yuan, Xiaoyan Jin, Zizhong Liu, Dingsheng Zhao, Youyou Li, Wenjuan Xing, Yuanyuan Fan, Zifan Liu, Junjie Pan, Zhen Zhen, Yunzhang Zhao, Qinna Yang, Jianwei Li, Yan-Zhong Chang, Yingxian Li

**Affiliations:** ^1^ Laboratory of Molecular Iron Metabolism, Key Laboratory of Molecular and Cellular Biology of Ministry of Education, College of Life Science, Hebei Normal University, Shijiazhuang, China; ^2^ State Key Laboratory of Space Medicine Fundamentals and Application, China Astronaut Research and Training Center, Beijing, China

**Keywords:** osteoblast derived exosomes, miR-21, hematopoietic injury, apoptosis resistance, irradiation

## Abstract

As hematopoietic stem cells can differentiate into all hematopoietic lineages, mitigating the damage to hematopoietic stem cells is important for recovery from overdose radiation injury. Cells in bone marrow microenvironment are essential for hematopoietic stem cells maintenance and protection, and many of the paracrine mediators have been discovered in shaping hematopoietic function. Several recent reports support exosomes as effective regulators of hematopoietic stem cells, but the role of osteoblast derived exosomes in hematopoietic stem cells protection is less understood. Here, we investigated that osteoblast derived exosomes could alleviate radiation damage to hematopoietic stem cells. We show that intravenous injection of osteoblast derived exosomes promoted WBC, lymphocyte, monocyte and hematopoietic stem cells recovery after irradiation significantly. By sequencing osteoblast derived exosomes derived miRNAs and verified *in vitro*, we identified miR-21 is involved in hematopoietic stem cells protection via targeting PDCD4. Collectively, our data demonstrate that osteoblast derived exosomes derived miR-21 is a resultful regulator to radio-protection of hematopoietic stem cells and provide a new strategy for reducing radiation induced hematopoietic injury.

## Introduction

Overdose radiation exposure caused various damage to the organism, including the hematopoietic system, gastrointestinal tract, kidney, skin and lung (Arora et al., 2010; Williams et al., 2010). Given that bone marrow is one of the most sensitive tissues to radiation, radiation exposure can result in bone marrow failure which shows up as hematopoietic stem cells (HSCs) decreased and self-renewal and differentiation impaired (Kato et al., 2013; Chang et al., 2015). For the surviving HSCs can replenish themselves and differentiate into blood cells of all hematopoietic lineages, reversing damage to hematopoietic stem cell is meaningful in clinical therapy after radiation.

Hematopoietic stem cells (HSCs) reside in specialized microenvironments that can regulate HSC fate in homeostasis or after injury stress (Mendelson and Frenette, 2014). Within these specialized microenvironments, the cells consisted of mesenchymal stromal cells, osteoblasts and other cells communicate with HSCs and adjust HSCs self-renewal and differentiation through either direct cell-to-cell contact or secretion of soluble factors (Doan and Chute, 2012; Ding and Morrison, 2013; Doan et al., 2013; Himburg et al., 2017). Exosomes, a kind of membrane-derived vesicles ranging in size from 30 to 150 nm, are considered as another mechanism of cell-to-cell communication and can alter the function of either nearby cells or distant cells through transfer of their cargo, which consist of proteins, lipids, and nucleic acids such as mRNA and non-coding small regulatory microRNAs (Lee et al., 2012; Flamant and Tamarat, 2016; Pitt et al., 2016; Xu et al., 2016). More recently, exosomes that are derived from mesenchymal stem cells have been implicated in hematopoietic regeneration after radiation injury (Wen et al., 2016; Schoefinius et al., 2017). The proposed mechanisms for that were their ability to increase expression of anti-apoptotic genes, downregulate pro-apoptotic genes, and stimulate pro-angiogenic effects by shuttling vascular endothelial growth factor (VEGF), insulin growth factor-1 and basic fibroblastic growth factor (Choi et al., 2014). Whether exosomes from other cell sources in hematopoietic microenvironments could contribute to hematopoietic regeneration is unknown.

Osteoblasts (OBs) are bone-forming cells that secrete calcium and synthesize the bone matrix. OBs cover the endosteal bone surface, forming an interface between calcified bone and marrow cells. OBs are reported to provide signals required for HSC quiescence, long-term maintenance, and BM retention (Calvi et al., 2003; Arai and Suda, 2007; Shiozawa et al., 2008). Visnjic et al. showed that ablation of OBs leads to loss of BM cellularity, decreased numbers of HSCs and progenitors in the BM, and increased extramedullary hematopoiesis (Visnjic et al., 2004). Calvi et al. reported that OBs are a regulatory component of the HSC niche *in vivo* that influences HSC function through Notch activation (Calvi et al., 2003). While, the role of osteoblast derived exosomes (OB-exosomes) in HSC self-renewal and regeneration remains unclear. Here, with primary sources of osteoblast cells to generate exosomes, we investigate the effects of OB-exosomes on hematopoietic regeneration after ionizing radiation.

In this study, we revealed the protective effect of OB-exosomes on radiation induced HSCs injury *in vitro* and *in vivo*. Then we identified miR-21 as an essential component in OB-exosomes in this protection effect by targeting the apoptosis inducing gene PDCD4. Taken together, our study revealed a new mechanism in which OB-exosomes mediated HSC protection through transferring miR-21 to downregulate PDCD4 expression which provided a potential therapeutic strategy against radiation induced bone marrow failure.

## Materials and Methods

### Cell and Culture Medium and Reagents

For the culture of osteoblast progenitor cells, calvariae from newborn mice were dissected aseptically and treated with 0.2% collagenase. The cells were maintained in the minimum essential medium (MEM) alpha containing 10% FBS. FDC-P1 cell line (ATCC, Manassas, VA, United States) was cultured in Dulbecco’s modified Eagle’s medium with 10% fetal bovine serum (FBS)/1% penicillin/streptomycin (PS).

### FDC-P1 Cell Line Irradiation

FDC-P1 cells were irradiated using an ELEKTA Compact medical accelerator 6 MV (Elekta, Stockholm, Sweden) at room temperature. 2 Gy radiation is the standard dose used in fractionated radiotherapy. A single dose of 2 Gy was delivered to the cells, with 100 cm source-to-skin distance and a dose rate of 2 Gy/min.

### Analysis of Apoptosis

Apoptotic cells were detected using the PE Annexin V apoptosis Detection Kit (BD Biosciences). EVs isolation and characterization.

When preparing culture media for vesicle collection or vesicle-cell co-culture, vesicle-depleted FBS (overnight ultracentrifugation at 100,000 g) was used. OBs were cultured in medium with exosome-depleted FBS for 3 days. Only less than eight passages of OBs were used to produce OB-exosomes. Unless otherwise noted, all vesicle separations in this study were by differential centrifugation at 300 *g* for 10 min, 2000 g for 30 min, 10,000 g for 1 h and 100,000 g for 1 h with collection of the exosomes. The exosome pellet was resuspended in PBS at an appropriate volume. Exosomes were used within 1 week after harvested for the *in vivo* studies. Exosomes functional effects *in vitro* were maintained for up to 6 months when stored in 10% DMSO at −80°C. The concentration of exosome suspension was measured by a BCA protein assay kit (Beyotime, China). The characterization of exosomes was confirmed by measuring morphology with a HT7700 (Hitachi, Japan) transmission electron microscope (TEM) and particle size with LM10 NanoSight Tracking analysis (Malvern Instruments, Britain).

### Analysis of Exosome Uptake by FDC-P1

The fluorescent dye 3,3′-dioctadecy loxacar bocyanine perchlorate (Dio) (5 μM, Invitrogen, United States) was added to exosome suspension, which was incubated for 15 min at 37°C. The pellet was washed twice with PBS followed by ultracentrifugation at 120,000 g for 1 h and then resuspended in PBS at an appropriate volume. FDC-P1 were incubated with the Dio-labeled exosomes for 4 h at 37°C. Next, the cells were washed twice with PBS and incubated with Hoechst 33,342 for 5 min at 37°C. The labeled cells were examined using confocal laser scanning microscope (LSM-710, Zeiss, Germany) and flow cytometry.

### *In Vivo* and *in Vitro* Exosome Treatment

*In vitro*, 15 μg/ml exosomes were added to 2 × 10^5^ FDC-P1. For *in vivo* treatment, 100 μg exosomes were intravenously injected into 8-week-old male C57BL/6 J mice at 1st,3rd,5th day after irradiation. In the control group, PBS was used.

### Western Blot

Total protein of cells was extracted in lysis buffer (50 mM Tris, pH 7.5, 250 mM NaCl, 0.1% SDS, 2 mM dithiothreitol, 0.5% NP-40, 1 mM PMSF and protease inhibitor cocktail) at 4°C for 30 min. Protein fractions were collected by centrifugation at 12,000 g, 4°C for 10 min. Next, 10 μg protein was subjected to SDS-PAGE and transferred onto polyvinylidene fluoride membranes. The membranes were blocked with 5% BSA and incubated with specific antibodies overnight. A horseradish peroxidase–labeled secondary antibody was added and visualized using an enhanced chemiluminescence kit (Pierce, United States). The following antibodies were used: TFIIB (1:1,000, CST, United States), Lamin A/C (1:1,000, CST, United States), β-actin (1:1,000, CST, United States), HSP70 (1:1,000, CST, United States), TSG101 (1:1,000, Santa Cruz Biotechnology, United States), Alix (1:1,000, CST, United States), PARP (1:1,000, CST, United States), BAX (1:1,000, CST, United States), PDCD4 (1:1,000, CST, United States) to examine the concentrations of proteins in the lysates, respectively.

### Animal Experiments

All mice were bred and housed in specific pathogen-free conditions under controlled temperature (22 ± 1°C) and exposed to a constant 12 h light-dark cycle in the animal facilities at China Astronaut Research and Training Center. 8-week-old C57BL/6 N male mice were used for the overall experiments in this study. Animal experiments were conducted in accordance with the guidelines and with the approval of the Committees of Animal Ethics and Experimental Safety of China Astronaut Research and Training Center.

### TBI and OB-Exosomes Injection

The mice were randomly divided into control group, 5 Gy TBI + PBS group and 5 Gy TBI + OB-exosomes group. The mice were exposed to TBI with γ-rays at a dose of 0.99 Gy/min. The mice received 100 μg of OB-exosomes by intravenous injection immediately at 1^st^,3^rd^,5^th^ day after irradiation. In the control group, PBS was used. The mice were euthanized on the 21st after TBI.

### Peripheral Blood Cell Counts

Blood was obtained from the mice via the orbital sinus and collected in 1.5 ml tubes coated with EDTA. K3. Peripheral blood cell counts (PBC) were calculated with a hematology analyzer (Orphee mythic220t).

### Bone Marrow Cell Isolation

For BM cells, freshly dissected femurs and tibias were flushed with PBS containing 2% fetal bovine serum. Then, the red blood cells were removed using Whole blood red cell lysing reagent. Bone marrow aspirates were passed through 70 µm pore strainers for isolation of bone spicules. Then, the strained bone marrow aspirates were diluted with an equal volume of phosphate‐buffered saline (PBS) and centrifuged over for 5 min at 500 g. Next, BM cells were resuspend using PBS supplemented with 2% fetal bovine serum.

### Flow Cytometry Analysis

For BM cells, freshly dissected femurs and tibias were flushed with PBS containing 2.5% fetal bovine serum. Red blood cells (RBC) were lysed with ammonium chloride–potassium bicarbonate lysis buffer. Lineage-negative (Lin-) stem-cell antigen (Sca-1)+c-kit+ (LSK) cells, long-term HSCs (LT-HSCs), short-term HSCs (ST-HSCs), and multipotent progenitors (MPPs) were identified based on cell-surface staining using biotin-conjugated lineage markers CD4 (RM4-5), CD8 (53–6.7), B220 (RA3-6B2), CD11c (N418), Ly-6G (RB6-8C5), TER119 (TER119), NK1.1 (PK136), and CD11b (M1/70) and then stained with Sca-1–peridinin chlorophyll protein–cyanine 5.5 (D7; Thermo Fisher Scientific), c-Kit–PE-Cy7 (2B8; Thermo Fisher Scientific), fms-related tyrosine kinase 3 (Flt3)–APC (A2F10; Thermo Fisher Scientific), CD34-FITC (RAM34; Thermo Fisher Scientific), Labeling with biotin-conjugated antibodies was revealed using streptavidin-APC-Cy7 (Thermo Fisher Scientific).

### Exosomal MicroRNA Analysis

OBs-derived exosomes were isolated as described above，and RNA isolation was performed with the miRNeasy Mini Kit (Qiagen, Hilden, Germany). Total RNA was quantified by the NanoDrop ND-2000 (Thermo Scientific) and the RNA integrity was assessed using Agilent Bioanalyzer 2,100 (Agilent Technologies). The sample labeling, microarray hybridization and washing were performed based on the manufacturer’s standard protocols. Briefly, total RNA was dephosphorylated, denaturated and then labeled with Cyanine-3-CTP. After purification the labeled RNAs were hybridized onto the microarray. After washing, the arrays were scanned with the Agilent Scanner G2505C (Agilent Technologies). The known microRNAs (miRNAs) in the OBs-exosomes were identified and their expression patterns in different samples were analyzed by Shanghai OE Biotech (China).

### RNA Extraction and Real-Time PCR

Total RNA from OB-exosomes or cells was extracted with TRIzol Reagent (Invitrogen, United States) and reverse transcribed with PrimeScript RT reagent Kit (TaKaRa, China) following the manufacturer’s instructions, and Real-time PCR was performed using SYBR Premix Ex Taq II Kit (Takara, China). For the quantitation of miRNA, the amount and purity of RNA was estimated by quawell micro volume spectrophotometer (Quawell, United States). Subsequently, miRNA was transcribed to cDNA using the miScript II RT Kit (Qiagen, Germany). The cDNA were used for detecting miRNA expression by Q-PCR using the miScript SYBR Green PCR Kit with miScript Primer Assay (Qiagen, Germany). The relative expression level of miRNA was determined by the cycle number via Q-PCR. Real-time PCR was performed using SYBR Premix Ex Taq II Kit (Takara, China). stem-loop RT-PCR was used for the quantification of miR-21. U6 was used as a normalization control in miRNA measurements. The sequences of the primers are:

mmu-miR-3963 sense primer: TGT​ATC​CCA​CTT​CTG​ACA​C
mmu-miR-7032-5p sense primer: CCC​AGG​GTG​GTC​CCC​GAG​AGG​GTG​GT
mmu-miR-6366 sense primer: AGC​TAA​GGG​GCC​CGG​GGA​GCC​A
mmu-miR-6769b-5p sense primer: CCT​GGT​GGG​TGG​GGA​AGA​GC
mmu-miR-451a sense primer: AAA​CCG​TTA​CCA​TTA​CTG​AGT​T
mmu-miR-466i-5p sense primer: TGT​GTG​TGT​GTG​TGT​GTG​TG
mmu-miR-1224-5p sense primer: GTG​AGG​ACT​GGG​GAG​GTG​GAG
mmu-miR-6944-5p sense primer: GTG​AGA​GCG​GGG​GGA​GTG​GCA​AG
mmu-miR-6368 sense primer: CTG​GGA​AGC​AGT​GGA​GGG​GAG
mmu-miR-8110 sense primer: AAG​CGT​GGA​TTG​GGG​GGG​GGG
mmu-miR-5100 sense primer: TCG​AAT​CCC​AGC​GGT​GCC​CTC​T
mmu-miR-9-3p sense primer: ATA​AAG​CTA​GAT​AAC​CGA​AAG​T
mmu-miR-21a-5p sense primer: TAG​CTT​ATC​AGA​CTG​ATG​TTG​A
mmu-miR-574-5p sense primer: TGA​GTG​TGT​GTG​TGT​GAG​TGT​GT
mmu-miR-125b-5p sense primer: TCC​CTG​AGA​CCC​TAA​CTT​GTG​A
mmu-miR-5112 sense primer: TAG​CTC​AGC​GGG​AGA​GCA​C
mmu-miR-3082-5p sense primer: GAC​AGA​GTG​TGT​GTG​TCT​GTG​T
mmu-miR-1187 sense primer: TAT​GTG​TGT​GTG​TAT​GTG​TGT​AA
mmu-miR-22-3p sense primer: AAG​CTG​CCA​GTT​GAA​GAA​CTG​T
mmu-miR-486a-5p sense primer: TCC​TGT​ACT​GAG​CTG​CCC​CGA​G
mmu-miR-3095-3p sense primer: TGG​ACA​CTG​GAG​AGA​GAG​CTT​TT
mmu-miR-2137 sense primer: GCC​GGC​GGG​AGC​CCC​AGG​GAG
mmu-miR-6908-5p sense primer: TTG​CCT​GTC​AGG​GAG​AGC​CCC​A
mmu-miR-7082-5p sense primer: TAC​GGG​CAG​GAG​GAG​GGG​AGG
mmu-miR-3085-5p sense primer: AGG​TGC​CAT​TCC​GAG​GGC​CAA​GAG​T
mmu-miR-374b-3p sense primer: GGT​TGT​ATT​ATC​ATT​GTC​CGA​G
mmu-miR-3960 sense primer: GGC​GGC​GGC​GGA​GGC​GGG​GG
mmu-miR-7036a-5p sense primer: AGC​GGG​GTT​CGG​TGG​GGA​AGA​GA
mmu-miR-7118-5p sense primer: TGG​GGA​AGG​CGG​GAG​AGG​GAA​C
mmu-miR-7011-5p sense primer: AGG​AGG​ATG​GGA​GAG​GGA​GGT​GT
General anti-sense primer: GAA​TCG​AGC​ACC​AGT​TAC​GC
mmu-U6 sense primer: CGC​TTC​GGC​AGC​ACA​TAT​A
mmu-U6 anti-sense primer: TTC​ACG​AAT​TTG​CGT​GTC​AT.

### MiRNA Function Study

The function of miRNA-21 in affecting the apoptosis of FDC-P1 was studied with the miRNA mimics (GenePharma). The apoptosis of FDC-P1 treated with miRNA mimics was assessed by flow cytometry and western-blot.

### miRNA Mimic Transfection

Cells for RNA interference were transfected with miR-21 mimics, control siRNAs (GenePharma) at 60% using Lipofectamine RNAiMAX in OptiMEM as per the manufacturer’s instructions (Invitrogen). MiR-21 mimics were synthesized by Gene Pharma (Shanghai, China). The sequences were listed as follow: miR-21 mimics sequences: 5′- UAG​CUU​AUC​AGA​CUG​AUG​UUG​A-3′; control: 5′-UUC​UCC​GAA​CGU​GUC​ACG​UTT-3′.

### Statistical Analysis

All experiments are presented as the mean ± SEM. Student’s t-test was used for statistical evaluations of two group comparisons. One-way analysis of variance (ANOVA) for more than two groups was performed for statistical analysis. All statistical analyses were performed with Prism software (GraphPad prism for windows, version 6.0).

## Results

### Osteoblast Derived Exosomes Promote White Blood Cell, Lymphocyte and Monocyte Recovery After Irradiation

**FIGURE 1:**
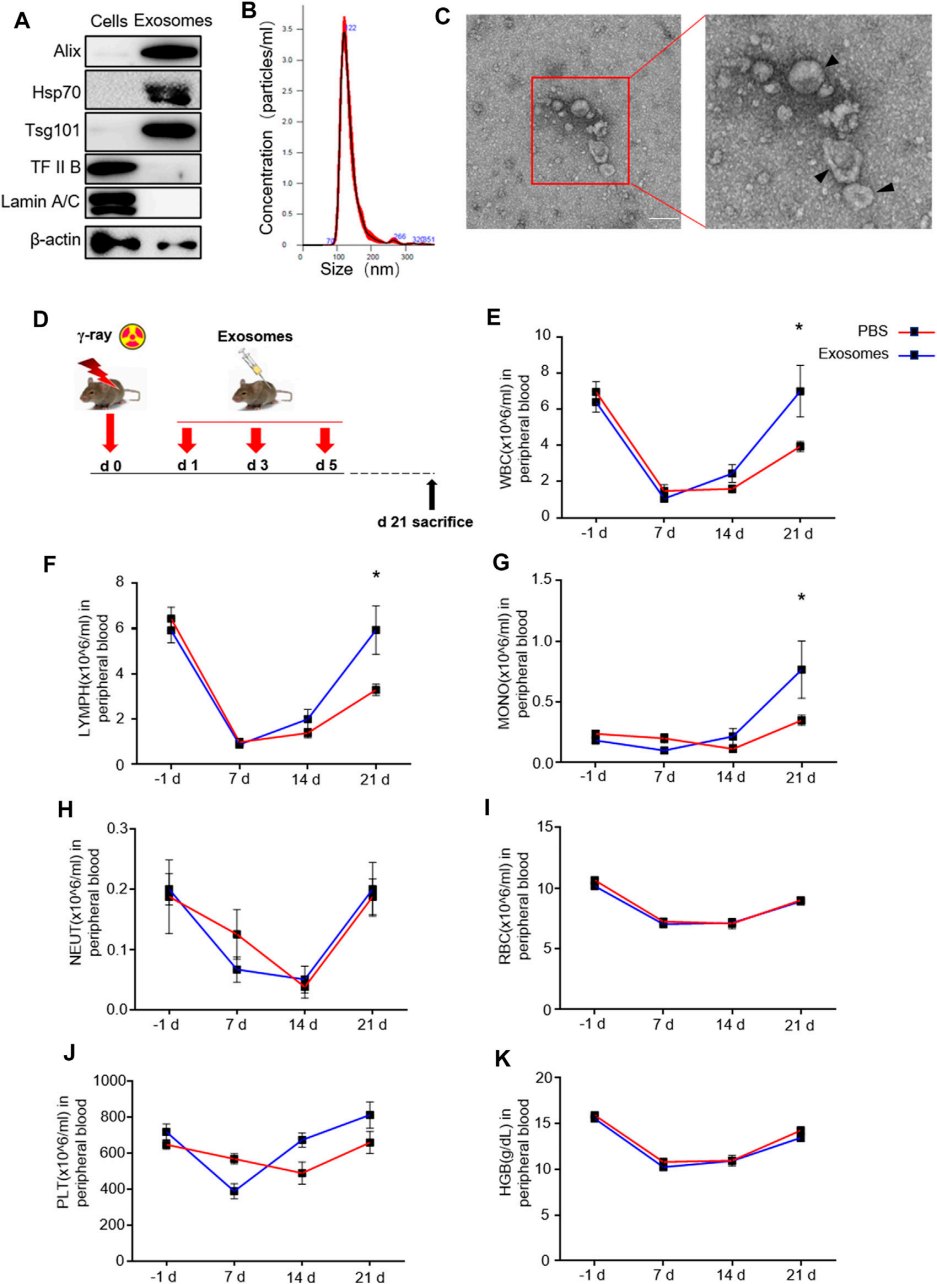
Osteoblast derived exosomes promote white blood cell, lymphocyte and monocyte recovery after irradiation. **(A)** Detection of protein levels of Alix, HSP70, TSG101, TFIIB and Lamin A/C in OB-exosomes and parental cell lysates by western blot. **(B)** Nanoparticle tracking the size distribution of OB-exosomes (representative of five independent measurements). **(C)** Representative transmission electron microscope (TEM) images of OB-exosomes. Scale bar, 200 nm. **(D)** The mice were injected with PBS or OB-exosomes immediately after 5 Gy TBI at 1st, 3rd, 5th day after irradiation. **(E)** white blood cell counts, **(F)** lymphocytes counts, **(G)** monocyte counts, **(H)** neutrophils counts, **(I)** red blood cell counts, **(J)** platelet counts and **(K)** hemoglobin level were measured by a hematology analyzer. Statistical analysis of peripheral blood cell number in irradiated mice injected with PBS (n = 8) and osteoblast-exosomes (*p < 0.05, mean ± sem, *n* = 6).

Because primary murine osteoblast derived exosomes (OB-exosomes) have not been previously generated, we sought to isolate and characterize these exosomes. As described in detail in the methods and materials section, exosomes were isolated from primary osteoblast cultures. Exosome markers Alix, HSP70, and TSG101 were enriched, while nuclear proteins TFⅡB and Lamin A/C were undetectable in OB-exosomes (Figure 1A). These data collectively verified that the purity of isolated exosomes was eligible for following experiments. Nanoparticle light scattering tracking and transmission electron microscopy analysis demonstrated that most of these exosomes were rounded vesicles and less than 150 nm in size (Figure 1B/C). Total body irradiation (TBI) can induce injury to self-renewal and differentiation of HSCs and make the decrease of white blood cell（WBC）numbers in the peripheral blood. Next, we measured the number of WBC, lymphocyte (LYMPH), monocyte (MONO), neutrophil (NEUT), red blood cell (RBC), platelet (PLT) and hemoglobin (HGB) with automatic Blood Cell Counter. On the 7th day after TBI, compared with that before TBI, a significant decrease in the number of WBCs was observed in mice radiated at a dose of 5 Gy, along with a decrease in the LYMPH, NEUT, RBC, PLT and HGB, which reflected impaired HSC differentiation. OB-exosomes injection improved the numbers of WBC, LYMPH and MONO in radiated mice on the 21st day after TBI (Figures 1E–K). These data indicate that OB-exosomes promote WBC, LYMPH and MONO recovery after irradiation.

### Osteoblast Derived Exosomes Mitigate Irradiation Injury to Hematopoietic Stem and Progenitor Cells

Hematopoietic stem and progenitor cells were detected and characterized using surface markers, including a lineage mixture (Ter119, CD11b, Gr1, B220, TCRgd, NK1.1, CD3e, CD11c), Sca1, and c-Kit, CD34, CD135.

**FIGURE 2:**
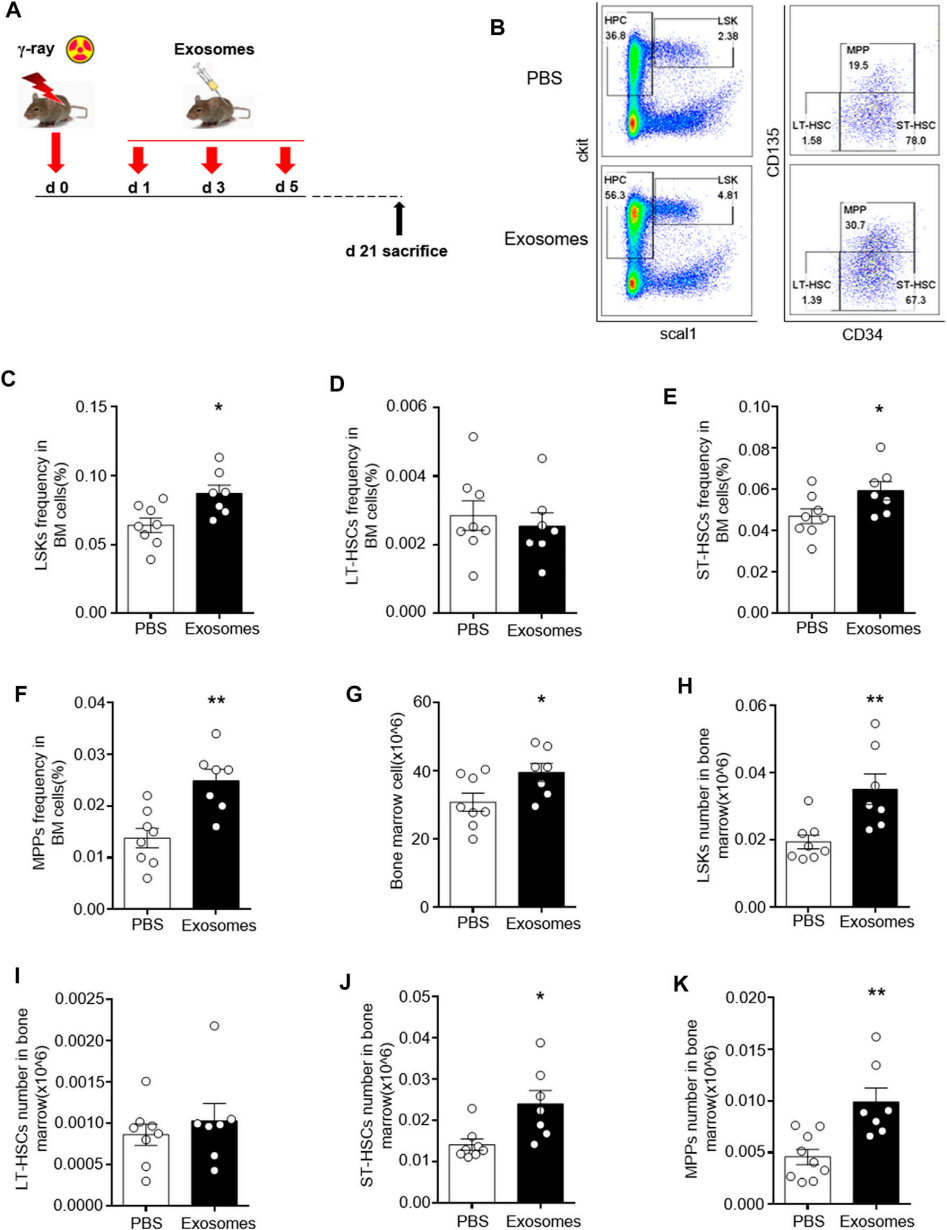
Osteoblast derived exosomes mitigate irradiation injury to hematopoietic stem and progenitor cells. **(A)** The mice were injected with PBS or OB-exosomes immediately after 5 Gy TBI at 1st, 3rd, 5th day after irradiation. **(B)** Representative FACS plots of LSK cells, MMP, LT-HSC and ST-HSC were detected by flow cytometry. **(C)** The LSK frequency, **(D)** LT-HSC frequency **(E)** ST-HSC frequency, **(F)** MPP frequency, **(G)** BM numbers, **(H)** LSK numbers, **(I)** LT-HSC numbers, **(J)** ST-HSC numbers, **(K)** MPP numbers were analyzed 21 days after 5 Gy TBI. Statistical analysis of the hematopoietic stem cell number in irradiated mice injected with PBS (*n* = 9) and osteoblast-exosomes (*p < 0.05, **p < 0.01, mean ± sem, *n* = 7).

We defined lineage-negative, Sca1-positive, and c-kit-positive cells (Lin^−^Sca1^+^c-Kit^+^) as LSK cells, which represent HSCs. Within the LSKs population, the CD34-negative part represents long-term HSCs, the CD34-positive, CD135-negative part represents short-term HSCs, and the double positive part represent multipotential progenitor cell (MPP). We detected the percentages of the above cells by flow cytometry, as shown in Figure 2B. Decreases in LSK cells, LT-HSC, SH-HSC and MPP were found in mice exposed to 5 Gy TBI. However, significant improvements in all these injuries in hematopoietic stem and progenitor cells were observed in radiated mice injected with OB-exosomes (Figures 2C–K). These data indicate that OB-exosomes injection reduce HSC injury caused by irradiation *in vivo*.

### Osteoblast Derived Exosomes Inhibit Irradiation Induced Apoptosis of FDC-P1

**FIGURE 3:**
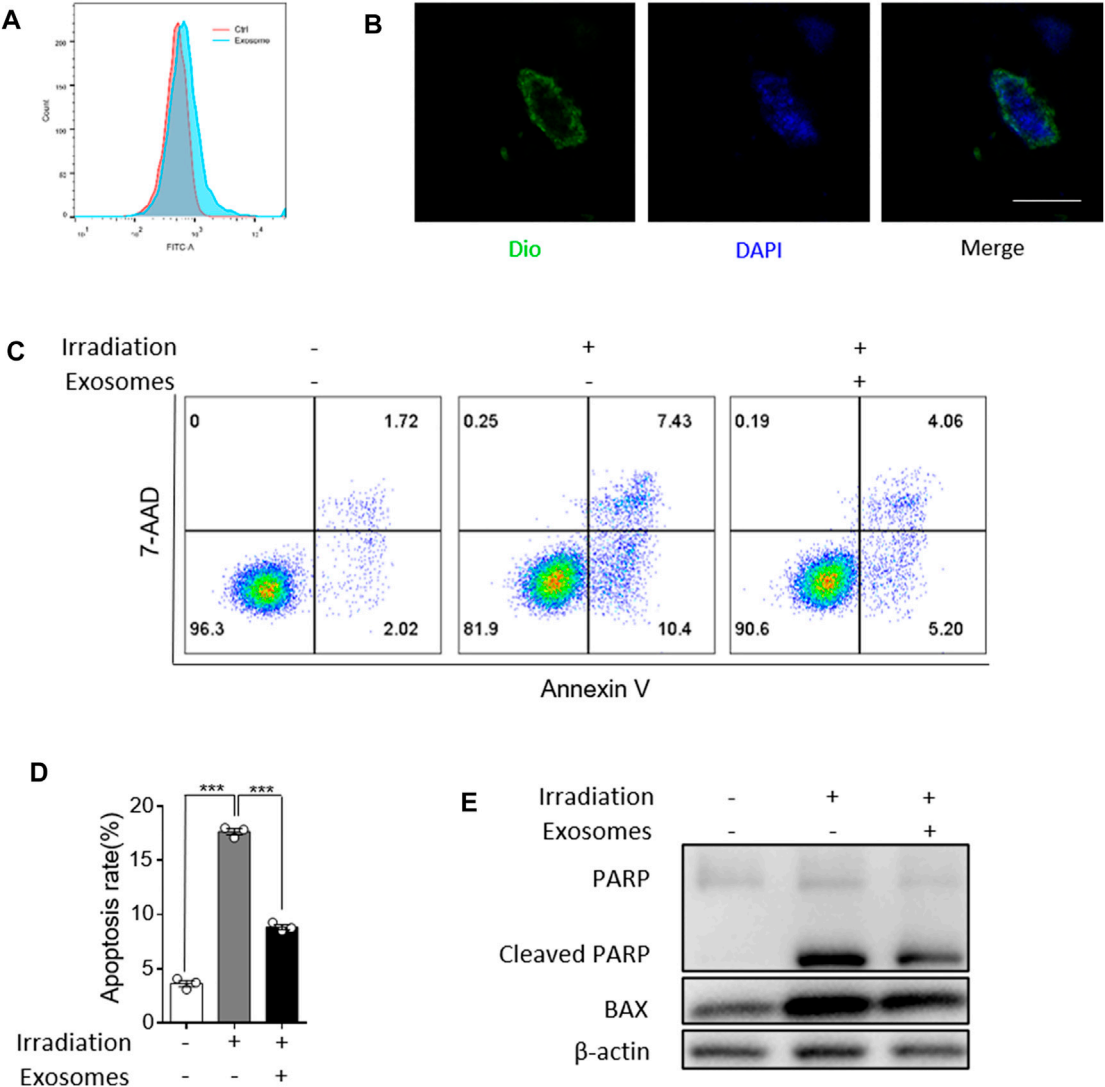
Osteoblast derived exosomes inhibit irradiation induced apoptosis of FDC-P1. **(A,B)** Representative imaging showed the fluorescence intensity signal detected by flow cytometry. Colocalization of OB-exosomes with FDC-P1 cells using confocal microscopy imaging. Exosomes were labeled by 3′-dioctadecyloxacarbocyanine perchlorate (Dio, green) and cell nuclei were stained with DAPI (blue). Scale bar, 10 μm. **(C)** FDC-P1 cells were irradiated with 2 Gy before treated with OB-exosomes, and apoptosis was measured by flow cytometry. *n* = 3/group **(D)** Statistical analysis of the difference between control, irradiation and co-cultured with OB-exosomes after irradiation groups (***p < 0.001, mean ± sem, *n* = 3/group). **(E)** Detection of protein levels of Cleaved PARP and BAX in irradiated FDC-P1 by western blot.

We next analyzed the effect of OB-exosomes on irradiated FDC-P1 cells, a mouse bone marrow cell line, to determine whether OB-exosomes could inhibit the apoptosis after irradiation *in vitro*. Firstly, Flow cytometry and Confocal imaging showed that Dio-labelled exosomes could be incorporated into FDC-P1 (Figure 3A/B). Then, FDC-P1 cells were irradiated with 2 Gy X-Ray and treated with OB-exosomes (15 μg/ml) for 24 h. The apoptotic cells were measured by flow cytometry (Figure 3C). The results showed that the percentage of apoptotic cells in the control group was 3.7 ± 0.6% and the irradiated group was 17.8 ± 0.8%, while the irradiated and OB-exosomes group was 8.7 ± 0.5% (Figure 3D). The results indicated that OB-exosomes treatment could inhibit the apoptosis of FDC-P1 cells induced by radiation. We next investigated if OB-exosomes exposure could decrease the elevation of radiation-induced apoptosis-associated protein. We analyzed the protein levels of BAX and cleaved PARP by western blot. After exposed to 2 Gy irradiation, FDC-P1 cells were cultured with or without OB-exosomes (15 μg/ml) for 24 h. There was a significant increase in cleaved PARP and BAX protein levels after radiation exposure. As expected, OB-exosomes treatment significantly reduced the level of cleaved PARP and BAX (Figure 3E), indicating that apoptosis-associated protein in irradiated cells was reversed with OB-exosomes treatment.

### Analysis the miRNA in Osteoblast Derived Exosomes

**FIGURE 4:**
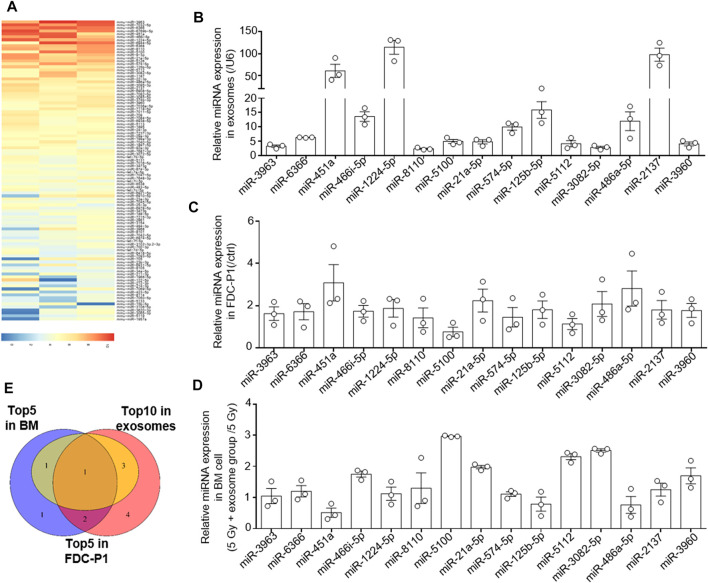
The osteoblast derived exosomes miRNA analysis. **(A)** The heat map of miRNA in OB-exosomes, the red color represents high miRNA expression level, and the blue color represents low miRNA expression level. **(B)** qRT-PCR analysis of expression of miRNA levels in OB-exosomes. **(C)** qRT-PCR analysis of expression of miRNA levels in FDC-P1 treated with or without OB-exosomes for 6 h. The relative expression of miR-21 was normalized to U6. **(D)** qRT-PCR analysis of expression of miRNA levels in bone marrow cells of irradiated mice injected with or without OB-exosomes. (mean ± sem, *n* = 3). **(E)** Venn diagram of top 10 miRNA in OB-exosomes, top 5 miRNA in FDC-P1 and top 5 miRNA in bone marrow.

We further investigated the mechanism by which OB-exosomes inhibited apoptosis in HSCs. miRNAs are one of the key molecules in exosomes to modulate recipient cells’ function, thus, we used microarray to identify miRNAs in OB-exosomes (Figure 4A). Among microRNAs contained in OB-exosomes, we identified the top 30 miRNAs in microRNA microarray and detected the top 30 miRNAs based on the expression level in OB-exosomes by qPCR (Figure 4B). Based on these results, levels of the top 15 miRNAs enriched in OB-exosomes were further analyzed in FDC-P1 treated with or without OB-exosomes. miR-21–5p, miR-451 and miR-486–5p exhibited the significant changes with more than 2-fold upregulation compared with control (Figure 4C), while the level of pre-miRNA was not altered (data not shown), suggesting that the increased miRNA in FDC-P1 was transported from exosomes, but not an intrinsically inducible expression. Furthermore, the miRNA expression changes in bone marrow cells were also analyzed after the irradiated C57BL/6 mice injected with OB-exosomes. miR-21-5p, miR-3082-5p, miR-5100 and miR-5112 exhibited the significant changes in bone marrow cells of radiated mice injected with OB-exosomes (Figure 4D). As shown in Figure 4E, only miR-21-5p was overlapped among the top 10 miRNAs in OB-exosomes, the top 5 miRNAs in bone marrow cells of radiated mice injected with OB-exosomes and the top 5 miRNAs in FDC-P1 treated with OB-exosomes. MiR-21 has been reported to be involved in anti-apoptotic effects (Xiao et al., 2016; Canfrán‐Duque et al., 2017; Cheng et al., 2018; Hu et al., 2020), and the function of miR-21 has been proved in related with hematopoietic apoptosis (Mengjia Hu et al., 2021). Above all, we focused on miR-21 to affirm the effect of OB-exosomes on HSC apoptosis.

### MiR-21 in Osteoblast Derived Exosomes Alleviated Irradiation Induced FDC-P1 Apoptosis

**FIGURE 5:**
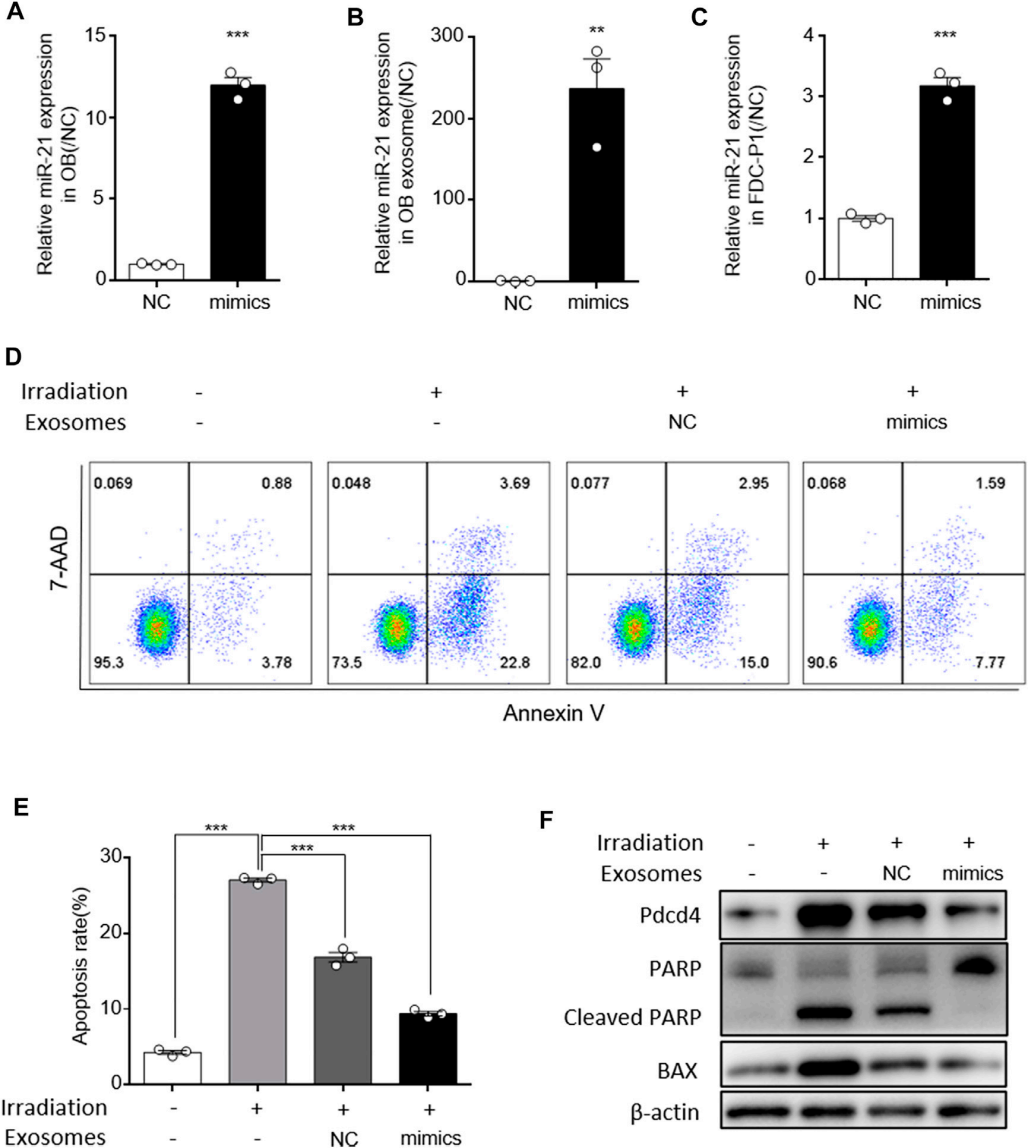
MiR-21 in osteoblast derived exosomes alleviated irradiation induced FDC-P1 apoptosis. **(A)** qRT-PCR analysis of expression of miR-21 levels in osteoblast transfected with NC or miR-21 mimics. **(B)** qRT-PCR analysis of expression of miR-21 levels in OB-exosomes transfected with NC or miR-21 mimics. **(C)** qRT-PCR analysis of expression of miR-21 levels in FDC-P1 which were cocultured with OB-exosomes transfected with NC or miR-21 mimics. **(D)** FDC-P1 cells were irradiated with 2 Gy before co-cultured with OB-exosomes (NC/miR-21 mimics), and apoptosis was measured by flow cytometry. **(E)** Statistical analysis of the difference between control, irradiation and co-cultured with OB-exosomes (NC/miR-21 mimics) after irradiation groups. (mean ± sem, *n* = 3/group). **(F)** FDC-P1 cells were irradiated with 2 Gy before co-cultured with OB-exosomes (NC/miR-21 mimics), and protein levels of Cleaved PARP and BAX was measured by western blot.

To further confirm the effect of exosomal miR-21 on HSC apoptosis, exosomes were isolated from osteoblasts cells transfected with miR-21 mimics. After transfected with miR-21 mimics, miR-21 levels were elevated in osteoblasts, and exosomes isolated from these osteoblasts showed higher miR-21 levels (Figures 5A,B). After treated with these exosomes, miR-21 levels in FDC-P1 were upregulated compared with control (Figure 5C). When irradiated FDC-P1 were incubated with exosomes secreted by osteoblast cells transfected with miR-21 mimics, the effect on reducing the percentage of apoptotic cells was reinforced (Figures 5D,E). We next investigated the effect of the miR-21-high exosomes on decreasing the elevation of radiation-induced apoptosis-associated protein. We treated irradiated FDC-P1 cells with miR-21-high exosomes and control (15 μg/ml) for 24 h, then the protein levels of BAX and cleaved PARP were analyzed by western blot (Figure 5F). The results showed that the effect of radiation on reduced cleaved PARP and BAX was also reinforced. PDCD4, a reported direct target of miR-21 (Zhang et al., 2013), also serves as an important factor in apoptosis function. For this reason, the expression of PDCD4 was detected at the same time to confirm the miR-21 mediated the effect of OB-exosomes on irradiation induced apoptosis (Figure 5F). Compared with control, decreased PDCD4 expression were observed in FDC-P1 treated with miR-21-high exosomes. Collectively, these data suggest that miR-21 serves as an important mediator of the protective effect of OB-exosomes on FDC-P1 cell survival in irradiation.

## Discussion

**FIGURE 6:**
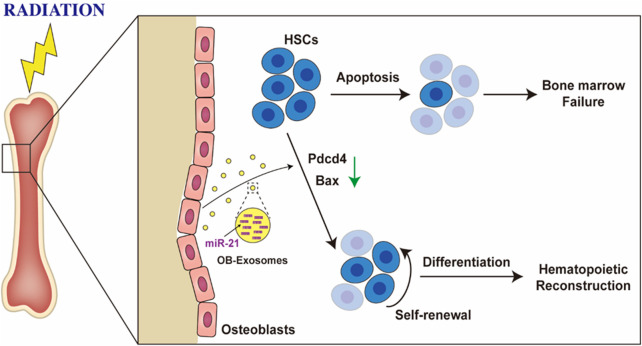
Osteoblast derived exosomes inhibit radiation-induced damage to hematopoietic stem cell. OB-exosomes led to a recovery of hematopoietic stem and progenitor cells numbers *in vivo* which decreased after total body irradiation, and significant improvement was observed in radiated mice injected with OB-exosomes. The miR-21 of OB-exosomes was identified as the target mediated the effect on the apoptosis of HSC.

In this study, we found that osteoblast derived exosomes are a novel regulator of hematopoietic stem cell self-renewal and differentiation. OB-exosomes led to a recovery of white blood cell, lymphocyte and monocyte numbers *in vivo* which decreased after total body irradiation, meanwhile, significant improvement on hematopoietic stem and progenitor cells was observed in radiated mice injected with OB-exosomes. Furthermore, OB-exosomes could inhibit the apoptosis of hematopoietic stem cell after irradiation *in vitro*. The miRNA of OB-exosomes was identified as the target mediated the effect on the apoptosis of HSC. MiR-21, which has been proved in related with hematopoietic apoptosis (Mengjia Hu et al., 2021), exhibited a significant change in bone marrow cells from radiated mice injected with OB-exosomes, and the result was further verified *in vitro*. The underlying mechanisms include the transfer of miR-21 between OB-exosomes and HSC, reduced PDCD4 protein levels, and downregulation of apoptosis related gene expression in HSC (Figure 6).

Radiation exposure can result in bone marrow failure which shows up as hematopoietic stem cells (HSCs) decreased and self-renewal and differentiation impaired (Kato et al., 2013; Chang et al., 2015; Guo et al., 2015; Henry and Arcangeli, 2021). For the importance of HSCs for self-renewal of the hematopoietic lineages and differentiation into blood cells, reversing damage to hematopoietic stem cell is meaningful in clinical therapy after radiation (Hoyer and Nielsen, 1992). As well known, bone marrow microenvironment is essential for HSC maintenance and kinds of paracrine mediators secreted by the neighboring cells exhibit protection on HSC function (Doan and Chute, 2012; Ding and Morrison, 2013; Doan et al., 2013; Himburg et al., 2017). Although, direct cell-to-cell contact or secretion of soluble factors can be used for clinical therapy to radiation, cell specificity and immunogenicity of cell therapy still need to be resolved. Exosomes which could bear nucleic acids such as mRNA and non-coding small regulatory microRNAs are considered as a novel regulator mediated cell-to-cell communications and can be used as a new approach for treatment of irradiation (Valadi et al., 2007; Lee et al., 2012; Flamant and Tamarat, 2016; Pitt et al., 2016; Xu et al., 2016). More recently, exosomes that are derived from mesenchymal stem cells have been implicated in hematopoietic regeneration after radiation injury (Wen et al., 2016; Schoefinius et al., 2017) and clearly reverse the radiation damage to marrow hematopoietic cells both *in vivo* and *in vitro* (Caplan and Dennis, 2006; Biancone et al., 2012; Bruno and Camussi, 2013). While the clinical use of MSC derived exosomes is restricted by the difficulty of the exosome quality control which caused by the cell heterogeneity of MSC, the secretion and inclusions of MSC derived exosome were significantly different under various stimulations. As candidate niche cells in the endosteal region, osteoblasts can support expansion of hematopoietic progenitor cells *in vitro* (Taichman et al., 1996; Calvi et al., 2003) and be correlated with the numbers of osteoblasts and LSK cells (Calvi et al., 2003; Zhang et al., 2003; Visnjic et al., 2004; Nilsson et al., 2005; Stier et al., 2005). Conditional ablation of osteoblasts induce acute alterations in hematopoiesis, including reduced numbers of lymphoid, erythroid and myeloid progenitors (Visnjic et al., 2004; Rauner et al., 2007), while the mechanism mediated the communication between osteoblasts and HSCs is still unclear. We sought to determine whether osteoblast cells (OBs)-derived exosomes could inhibit hematopoietic cells injury induced by radiation. Our results indicated that OB-exosomes injection alleviates TBI-induced HSC dysfunction, reduces HSC frequency injury in radiated mice. *In vitro*, co-culture with OB-exosomes could transfer into FDC-P1 cells and inhibit the apoptosis induced by radiation. The radiation-induced injury of hematopoietic stem cells can be suppressed by osteoblast-derived exosome-mediated cell-cell communication. In this study, we revealed the protective effect of OB-exosomes on radiation induced HSCs injury which could be used for therapy of irradiation injury.

Exosomes have the capacity to transfer biological information from parent cells to target cells (Valadi et al., 2007; Camussi et al., 2010; Lee et al., 2012; Xin et al., 2012; Pitt et al., 2016). Non-specific miRNA depletion of exosomes from Drosha knockdown-MSC was shown to abrogate the protective effect of exosomes in a kidney injury model (Collino et al., 2015), indicating that the miRNA in the exosomes may play a critical transcriptional role in the healing capacity of MSC. To explore the mechanisms of the osteoblast cells secreted exosomes in regulating HSC radiation resistance, we analyzed the miRNA profiles of exosomes derived from osteoblast cells. Base on the analysis of microRNAs contained in OB-exosomes by microarray, levels of the top 15 miRNAs enriched in OB-exosomes were further verified *in vitro*. 3 miRNAs exhibited the significant changes with more than 2-fold upregulation. Compared with the changes in bone marrow cells of radiated mice injected with OB-exosomes, only miR-21–5p was overlapped. The function of miR-21 has been proved in related with hematopoietic radiation damage resistance (Mengjia Hu et al., 2021). The anti-apoptotic effect of OB-exosomes overexpressing miR-21 was detected *in vitro*. Meanwhile, the PDCD4, a direct target of miR-21, also serves as an important factor in apoptosis function, was decreased (He et al., 2013; Fu et al., 2017; Wang et al., 2017; Xu et al., 2017; Feng et al., 2018). Our work showed that OB-exosomes overexpressing miR-21 could reinforced prevent radiation induced apoptosis in FDC-P1 cells, and reverse bone marrow cell radiation damage *in vivo*. This suggests that osteoblast derived exosomes mediate apoptosis prevention via a noncoding RNA-induced epigenetic alteration in FDC-P1. It is reported that bone marrow exposure to IR induced rapid depletion of hematopoietic stem cells and of their progenitors, while bone marrow mesenchymal stem cells can survive radiation. However, the surviving hematopoietic stem cells and osteoblast are senescent cells, which aggravate the dysfunction of hematopoietic stem cells self-renewal and differentiation and osteoblast function (Alessio et al., 2015; Alessio et al., 2017; Bai et al., 2020). In this study, we didn’t look at the role of miR-21 in the modulation of the senescence. However, our results demonstrated that miR-21 overexpressed exosomes treatment can protect irradiation induced hematopoietic stem cells apoptosis by inhibiting the mRNA levels of apoptosis gene and miR-21 inhibitor weakened the effect of exosome on irradiation induced apoptosis. These results suggested that miR-21 could be involved in the protection of renewed hematopoietic stem cells from senescence.

Osteoblast derived exosomes-mediated reversal of bone marrow stem cell damage caused by radiation suggests a unique new approach to radiation mitigation. Compared with HSC transplant, this exosomes-based, cell-free therapeutic strategy provides a convenient and safe therapeutic potential for rescuing marrow damage in patients treated with marrow toxic agents.

## Data Availability

The original contributions presented in the study are included in the article/Supplementary Material, further inquiries can be directed to the corresponding authors.

## Abbreviations

OB, osteoblast; MSC, mesenchymal stromal cell; TBI, Total body irradiation; BM, bone marrow; PBC, peripheral blood cell; WBC, white blood cell; LYMPH, lymphocyte; MONO, monocyte; NEUT, neutrophil; RBC, red blood cell, PLT, platelet; HGB, hemoglobin; HSC, hematopoietic stem cell; HSPC, hematopoietic stem progenitor cell; LSK, Lineage-negative (Lin-) stem-cell antigen (Sca-1)+c-kit+ cells; LT-HSC, long-term HSC; ST-HSC, short-term HSCs; MPP, multipotent progenitors cell; miRNAs, microRNAs; miR-21, miR-21a-5p; PDCD4, programmed cell death 4; DIO, 3,3′-dioctadecy loxacar bocyanine perchlorate; TEM, transmission electron microscope; exo, exosome.
