# Supplementary material for: Osteoblast Derived Exosomes Alleviate Radiation- Induced Hematopoietic Injury
Source: Front Bioeng Biotechnol. 2022 Apr 21;10:850303. doi: 10.3389/fbioe.2022.850303 (PMC9070646; doi:10.3389/fbioe.2022.850303)
Supplement: Supplementary file 1 [file DataSheet1.docx]

https://www.jianguoyun.com/p/DcUxbFoQ-rmYChjo0qgE
